# Transition-metal-catalyzed synthesis of phenols and aryl thiols

**DOI:** 10.3762/bjoc.13.58

**Published:** 2017-03-23

**Authors:** Yajun Liu, Shasha Liu, Yan Xiao

**Affiliations:** 1 College of Life Science and Medicine, Dalian University of Technology, Panjin 124221, China; 2 Department of Pharmaceutical Engineering, Shenyang Pharmaceutical University, Shenyang 110016, China

**Keywords:** aryl thiol, C–O bond, C–S bond, phenol, transition metal

## Abstract

Phenols and aryl thiols are fundamental building blocks in organic synthesis and final products with interesting biological activities. Over the past decades, substantial progress has been made in transition-metal-catalyzed coupling reactions, which resulted in the emergence of new methods for the synthesis of phenols and aryl thiols. Aryl halides have been extensively studied as substrates for the synthesis of phenols and aryl thiols. In very recent years, C–H activation represents a powerful strategy for the construction of functionalized phenols directly from various arenes. However, the synthesis of aryl thiols through C–H activation has not been reported. In this review, a brief overview is given of the recent advances in synthetic strategies for both phenols and aryl thiols.

## Introduction

**Figure 1:**
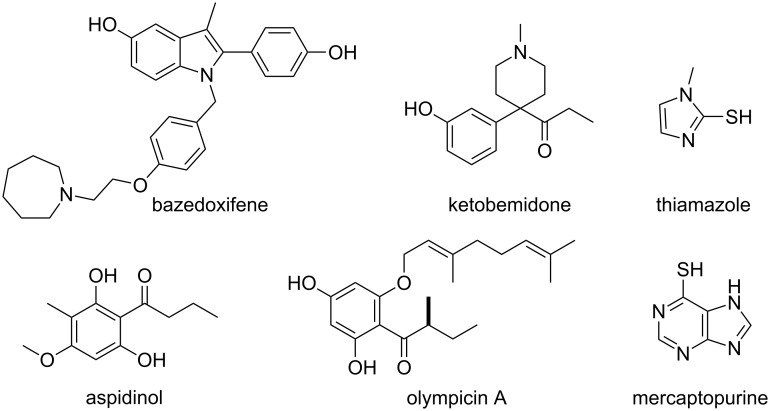
Examples of drugs bearing phenol or aryl thiol as central structural motifs.

Phenols and aryl thiols serve as both important intermediates in organic synthesis and also final products, playing important roles in pharmaceutical molecules, pesticides and polymers in both academia and industry [1–4]. Some classic drugs which employ phenols or aryl thiols as central structural motifs are shown in Figure 1.

In the past decade, the transition-metal-catalyzed Ullmann-type coupling reaction has emerged as an effective method, allowing the synthesis of phenols and aryl thiols from aryl halides through C–O and C–S bond formation, respectively [5–7]. Very recently, the C–H activation has made revolutionary advances in organic synthesis because it allows an access to functionalized products from simple arenes, avoiding their pre-functionalization [8–10]. The synthesis of phenols has greatly benefited from C–H activation, but the application in the synthesis of aryl thiols is still yet to be reported.

Both phenols and aryl thiols have similar chemical properties, such as acidity and nucleophilicity, and thus to some extent, the synthetic methods could be developed analogously to each other. In this context, it is valuable to compare their existing synthetic methods for better understanding, so as to inspire organic chemists to invent new methods for the synthesis of these two important classes of compounds. Herein, we review the recent developments on transition-metal-catalyzed syntheses of phenols from aryl halides and arenes, and aryl thiols from aryl halides.

## Review

### 1 Transition-metal-catalyzed synthesis of phenols

In early times, classic methods for the synthesis of phenols included the sulfonation of benzene [11], the Dakin reaction [12–13] and the Sandmeyer-type reaction [14]. These methods are useful for the preparation of various phenols, however, they suffer from several drawbacks such as multistep syntheses, toxic solvents, high temperatures, a narrow substrate scope, low selectivity, and/or low yields.

Inspired by the classic Ullmann reaction [15–16], the development of a transition-metal-catalyzed C–O coupling reaction provides various strategies to synthesize C–O-coupled products including ethers and phenols. On the other hand, the C–H hydroxylation, either with heteroatom-containing directing groups or without directing groups, has provided various methods for the synthesis of phenols. Compared with traditional methods, the transition-metal-catalyzed phenol synthesis has several advantages: broad substrate scope, mild reaction conditions, and easy access to starting materials.

#### 1.1 Aryl halide as substrate

Compared with other traditional starting materials, such as phenylsulfonic acid, aryl ketone and phenylboronic acid [17–20], aryl halides can be considered as simple and economical starting materials for the synthesis of phenols. In the beginning, palladium catalysts have attracted much attention due to their high conversion efficiency, and later copper catalysts, which are cheaper and more stable, have been extensively studied in this field.

**Scheme 1:**
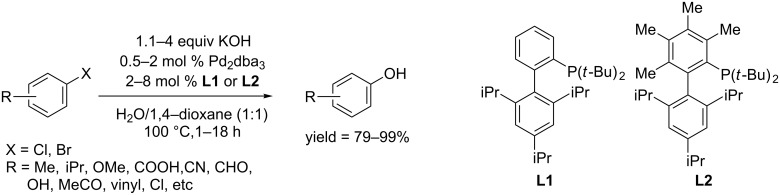
Hydroxylation of aryl halides using biphenylphosphine as ligand.

**1.1.1 Palladium-catalyzed hydroxylation of aryl halides:** In 2006, Buchwald and co-workers described the first synthesis of phenols from aryl halides through a palladium-catalyzed reaction [21]. The C–O coupling reaction of an aryl halide and potassium hydroxide took place when using Pd_2_dba_3_ as catalyst, biphenylphosphine (**L1** or the bulkier **L2**) as ligand, and 1,4-dioxane/H_2_O as solvent (Scheme 1). Under these conditions, a wide range of aryl bromides and chlorides could be readily converted to the corresponding phenols in high yields at 100 °C within 1–18 h. Moreover, sterically hindered *ortho*-functionalized aryl halides and heteroaryl halides were also converted into the corresponding phenols in excellent yields. Notably, they claimed that hydroxy salt was indispensable for the conversion as a replacement of KOH with K_3_PO_4_ afforded 99% yield of diaryl ethers.

**Scheme 2:**
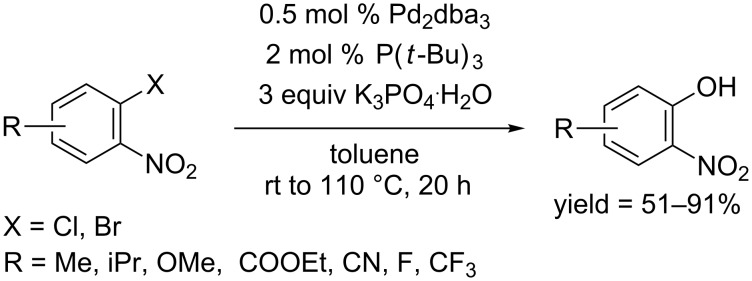
Hydroxylation of aryl halides using *tert*-butylphosphine as ligand.

Interestingly, the following work developed by the Kwong group in 2007 employed K_3_PO_4_ as the base and succeeded in the hydroxylation of aryl halides [22]. They chose tri-*tert*-butylphosphine as the ligand in their reaction system and obtained the phenols from aryl halides, suggesting a great influence of the ligand on the reaction performance (Scheme 2). However, their protocol was only suitable for activated halides having an *ortho*-nitro substituent; lower yields were observed in the case of unactivated aryl halides, such as 2-isopropylbromobenzene and 2,5-dimethylchlorobenzene.

**Scheme 3:**
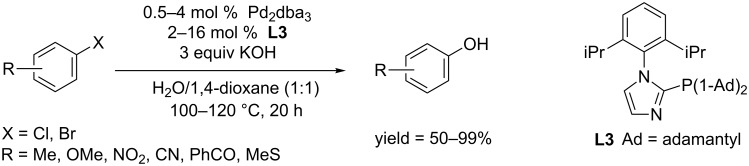
Hydroxylation of aryl halides using imidazole typed phosphine ligands.

Inspired by a huge effect of the phosphine ligand on the reaction performance, the Beller group synthesized a series of novel imidazole-based phosphine ligands, and their effciency was carefully screened [23]. Among these ligands, the one with two isopropyl groups located at the phenyl ring (**L3**) was effective in converting aryl chlorides and bromides to the corresponding phenols in moderate to excellent yields (Scheme 3).

**Scheme 4:**
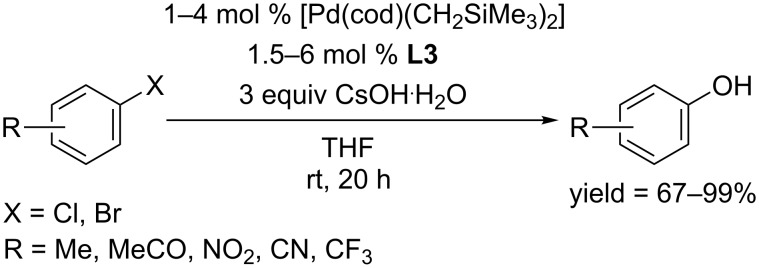
[Pd(cod)(CH_2_SiMe_3_)_2_] catalyzed hydroxylation of aryl halides.

The organopalladium complex was so effective that the reactions proceed even at room temperature. Based on their previous work, the Beller group studied all steps of the catalytic cycles and developed a combination of palladium precursor [Pd(cod)(CH_2_SiMe_3_)_2_] and **L3**, which allowed the reaction to proceed smoothly in THF at room temperature (Scheme 4) [24]. The reaction system used CsOH as base, providing phenols in moderate to excellent yields.

**Scheme 5:**
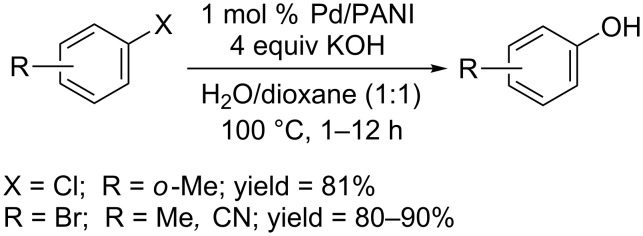
Pd/PANI catalyzed hydroxylation of hydroxylation of aryl halides.

In 2007, the Diaconescu group described Pd/PANI, which was prepared by supporting palladium nanoparticles with polyaniline (PANI) nanofibers. Pd/PANI catalyzed a Suzuki coupling reaction and hydroxylation of aryl halides [25]. The hydroxylation of aryl halides occurred at 100 °C in aqueous 1,4-dioxane in the presence of 1 mol % of Pd/PANI and 4 equiv of KOH (Scheme 5). Aryl bromide and iodides were converted to the corresponding phenols in good yields.

**Scheme 6:**
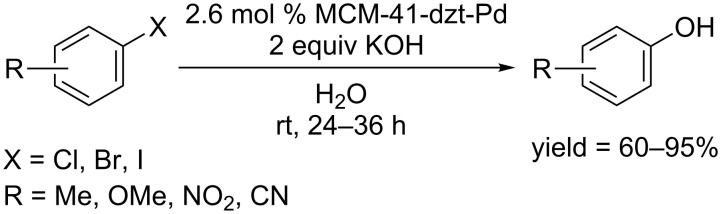
MCM-41-dzt-Pd catalyzed hydroxylation of aryl halides.

In 2015, Ghorbani-Choghamarani and co-workers designed a new type of nanocatalyst MCM-41-dzt-Pd through the immobilization of Pd(OAc)_2_ on the surface of dithizone(dzt)-functionalized mesoporous MCM-41 (Scheme 6) [26]. The developed catalyst was able to convert aryl halides to the corresponding phenols in water at room temperature. This catalyst can be reused many times without loss of the catalytic activity. Moreover, this catalyst could also be applied in the synthesis of anilines.

**1.1.2 Copper-catalyzed hydroxylation of aryl halides:** Copper catalysts are considered as economical and stable catalysts. However, copper catalysts often have a low catalytic activity to activate the C–Hal bond. Fortunately, along with the development of various bidentate ligands, the copper-catalyzed C–O coupling reaction has been extensively applied in the synthesis of phenols from aryl halides. However, the poorer efficiency of copper catalysts than that of palladium catalysts often limits the substrate scope to aryl iodides and aryl bromides.

**Scheme 7:**
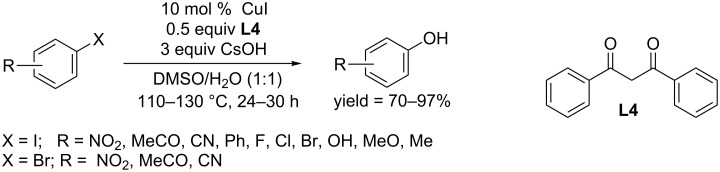
Hydroxylation of aryl halides using dibenzoylmethane as ligand.

In 2009, two independent works by Taillerfer and You opened the prelude to the copper-catalyzed hydroxylation of aryl halides. The Taillefer group found that a combination of CuI and bidentate ligand could promote the conversion of aryl halides to the corresponding phenols in a mixed solvent system of DMSO/H_2_O (1:1). Using iodobenzene as model substrate, various ligands were successfully tested: L-proline (yield 70%), 2-hydroxyacetophenone (yield 85%), *N*,*N*-dimethyl-3-oxobutanamide (yield 65%), tetramethylethylenediamine (TMEDA, yield 84%), phenanthroline (yield 75%), 2,2,6,6-tetramethyl-3,5-heptanedione (TMHD, yield 95%) and dibenzoylmethane (**L4**, yield 97%) [27–28]. The reaction system afforded phenols from aryl halides and aryl bromides bearing electron-withdrawing groups in moderate to excellent yields (Scheme 7). A broad scope of functional groups was tolerated. They also showed that electron-rich aryl bromides were also converted to phenols via a two-step procedure by addition of sodium iodide. A mechanistic study in the group of Jutand revealed the formation of a copper(I) complex from the 1,2-ketone and the hydroxy group, which further coupled with aryl iodides through oxidative addition to generate a copper(III) complex. Phenols were liberated by the following reductive elimination [29].

**Scheme 8:**
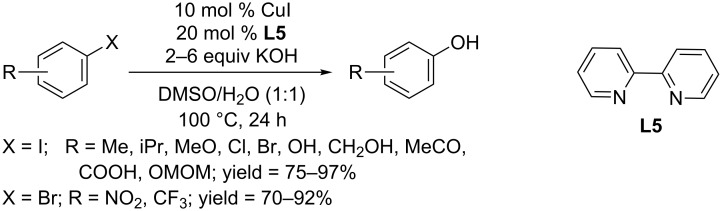
Hydroxylation of aryl halides using 2,2’-bipyridine as ligand.

You and co-workers used the same copper catalyst and reported that a *N*,*N*-bidentate ligand, 2,2’-bipyridine (**L5**) could prompt the conversion of aryl halides to phenols in the presence of KOH as coupling partner (Scheme 8) [30]. Aryl iodides and electron-deficient aryl bromides were easily converted to the corresponding phenols in good to excellent yields. A broad scope of functional groups including ether, halo, hydroxy, carboxylic acid and trifluoromethyl groups were well tolerated in their protocol. Even hindered substrates such as 2,6-diisopropyl iodobenzene also afforded the corresponding phenol in satisfying yield. The developed protocol was further applied in the synthesis of alkyl aryl ethers and benzofurans, which often possess interesting biological activities.

**Scheme 9:**
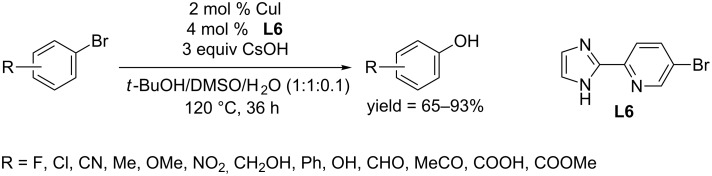
Hydroxylation of aryl bromides using imidazolyl pyridine as ligand.

In 2011, the Zhang and Ma group screened a series of 2-heteroarylpyridines and found another *N*,*N*-bidentate ligand, 5-bromo-2-(1*H*-imidazol-2-yl)pyridine (**L6**), and succeeded to synthesize phenols from aryl bromides in the presence of CuI as catalyst in the mixed solvent of *t*-BuOH–DMSO–H_2_O at 120 °C (Scheme 9) [31]. Both electron-rich and electron-deficient aryl bromides were converted to the corresponding phenols in good to excellent yields. Under the optimum conditions, switching the base CsOH to weaker KOH showed comparable yields.

**Scheme 10:**
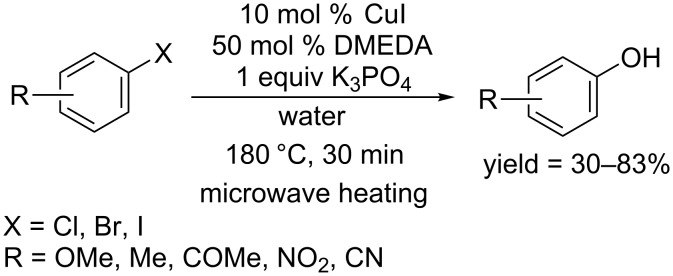
Hydroxylation of aryl halides using DMEDA as ligand.

In 2010, Leadbeater and co-workers used *N*,*N′*-dimethylethylenediamine (DMEDA) as ligand and performed the reaction using CuI as catalyst in the presence of K_3_PO_4_ [32] (Scheme 10). Under microwave heating, the reactions were accomplished within 30 min. A variety of aryl halides were converted to the corresponding phenols.

**Scheme 11:**
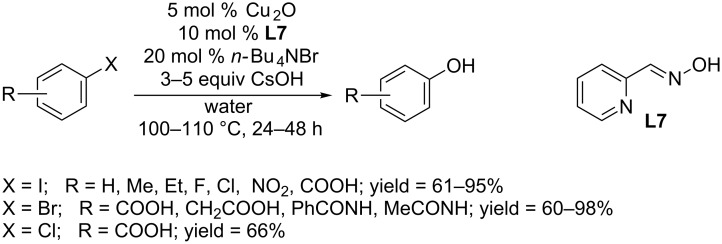
Hydroxylation of aryl halides using PAO as ligand.

Fu and co-workers employed pyridine-2-aldoxime (PAO, **L7**) as ligand and developed a Cu_2_O catalyzed hydroxylation protocol [33]. The reaction was carried out at 110 °C in water in the presence of *n-*Bu_4_NBr as phase transfer catalyst (Scheme 11). Aryl iodides and electron-deficient aryl bromides and chlorides were converted to the corresponding phenols. A broad range of functional groups are well tolerated under their optimum condition.

Besides the opening work using TMHD by the Taillefer group, other O,O-bidentate ligands also played important roles in C–O coupling reactions for the synthesis of phenols from aryl halides.

**Scheme 12:**
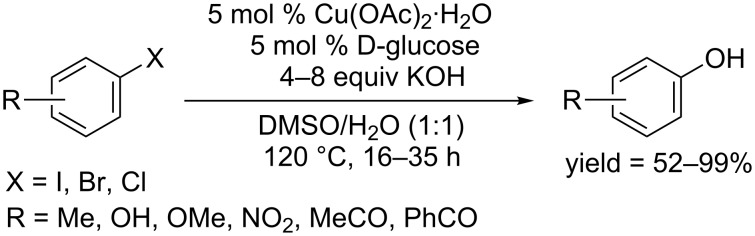
Hydroxylation of aryl halides using D-glucose as ligand.

In 2011, the Sekar group used D-glucose as ligand and reported a Cu(OAc)_2_ catalyzed synthesis of phenols from aryl halides in the presence of KOH in DMSO/H_2_O (1:1) at 120 °C (Scheme 12) [34]. Aryl iodides and electron-deficient aryl bromides provided good to excellent yields. D-Glucose represents a type of environmentally friendly ligand and can be easily removed during the work-up process. This work is of special value as it was the first report employing copper(II) as the catalyst in the synthesis of phenols.

**Scheme 13:**
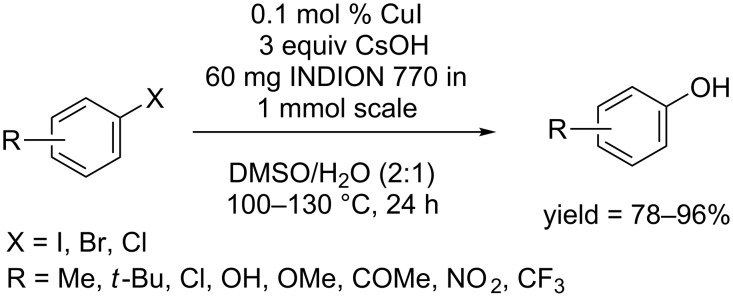
Hydroxylation of aryl halides using INDION-770 as ligand.

In 2011, the Maheswaran group used the sulfonic acid resin INDION-770 as additive and developed a CuI-catalyzed protocol for the hydroxylation of aryl halides in a DMSO/H_2_O (2:1) solvent system (Scheme 13) [35]. In this heterogeneous reaction system, the cation of CuI was attached to the sulfonic acid resin, and could be easily recovered and reused. Aryl iodides, activated aryl bromides and chlorides, and heteroaryl bromides were smoothly converted to phenols.

**Scheme 14:**
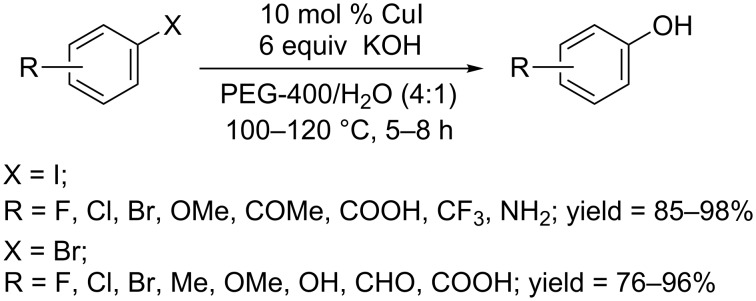
PEG-400 mediated hydroxylation of aryl halides.

In 2011, the Chen group developed a PEG-400-mediated protocol for the synthesis of phenols using CuI as the catalyst and KOH as the base (Scheme 14) [36]. In this reaction system, non-toxic and cheap PEG-400 played a dual role as both ligand and solvent. The effective catalytic system could convert aryl iodide to phenols in high yields within 5 hours at 100 °C. The conversion of aryl bromides bearing either an electron-donating group or an electron-withdrawing group required higher temperatures (120 °C) and a longer reaction time (8 hours).

**Scheme 15:**
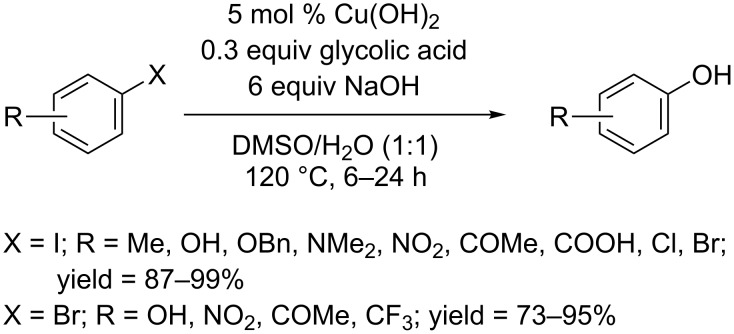
Hydroxylation of aryl halides using glycolic acid as ligand.

In 2013, the Chae group reported that a simple ligand, glycolic acid, could promote the conversion of aryl halides to phenols in the presence of Cu(OH)_2_ and NaOH in the solvent of DMSO/H_2_O (1:1) (Scheme 15) [37]. Aryl iodides can give the corresponding phenols in excellent yields. Typically, the *ortho*-bromo group was not affected under standard conditions for the conversion of aryl iodides. Electron-deficient aryl bromides were converted by increasing the catalyst loading to 10 mol % or extending the reaction time. Interestingly, a similar catalytic system using CuI as catalyst and Cs_2_CO_3_ as base predominantly afforded ethers rather than phenols.

**Scheme 16:**
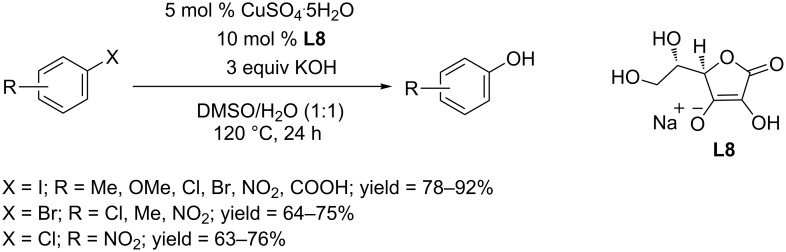
Hydroxylation of aryl halides using L-sodium ascorbate as ligand.

In 2015, the Wang group employed CuSO_4_·5H_2_O as catalyst and screened a series of ligands, revealing that L-sodium ascorbate (**L8**) could promote the synthesis of phenols (Scheme 16) [38]. The reaction occurred at 120 °C in the presence of KOH as base, converting aryl iodides and nitro-substituted aryl bromides and chlorides to the corresponding phenols in moderate yields.

**Scheme 17:**
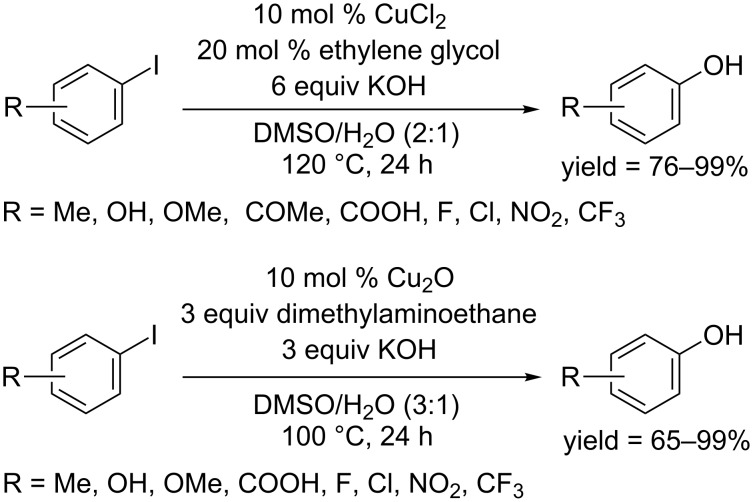
Difunctionalized ethanes mediated hydroxylation of aryl iodides.

Phenols can be obtained from phenyloxylethanols through an intramolecular nucleophilic substitution [39]. In 2015, the Chae group found difunctionalized ethanes including 2-dimethylaminoethanol and ethylene glycol can function as ligand to work with Cu(OAc)_2_ in the presence of KOH, affording phenols from aryl iodides in moderate to excellent yields (Scheme 17) [40].

**Scheme 18:**
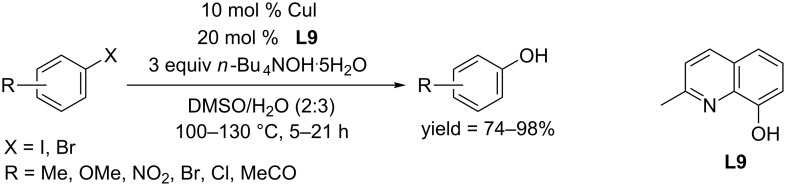
Hydroxylation of aryl halides using 2-methyl-8-hydroxylquinoline as ligand.

In 2010, a screening work for the ligand by the Punniyamurthy group led to the discovery of a new type of N,O-bidentate ligand, 2-methyl-8-hydroxyquinoline (**L9**), which could promote the CuI-catalyzed hydroxylation of aryl halides in the presence of *n*-Bu_4_NOH·5H_2_O without a strong base (Scheme 18) [41]. Remarkably, aryl bromides bearing either an electron-donating group or an electron-withdrawing group were converted to the corresponding phenols in excellent yields. In the same year, the Jiang and Ma group found 8-hydroxyquinoline could function as ligand in the hydroxylation of aryl iodides in the presence of CuI as catalyst and KOH as base [42].

**Scheme 19:**
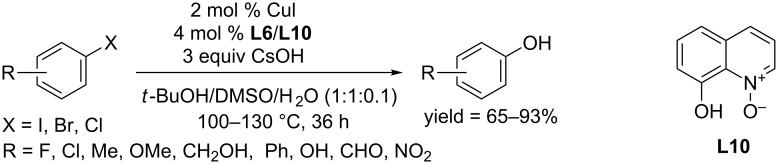
Hydroxylation of aryl halides using 8-hydroxyquinolin-*N*-oxide as ligand.

In 2011, the Jiang group employed 8-hydroxyquinoline-*N*-oxide (**L10**) as ligand and hydroxylation occurred in the presence of CuI as catalyst and CsOH as base (Scheme 19) [43]. The catalytic activity of the reaction system depended on the reaction temperature. The reactions of aryl iodides and aryl bromides were carried out at 100 °C and 110 °C, respectively. The conversion of aryl chlorides bearing electron-withdrawing groups was achieved at 130 °C.

**Scheme 20:**
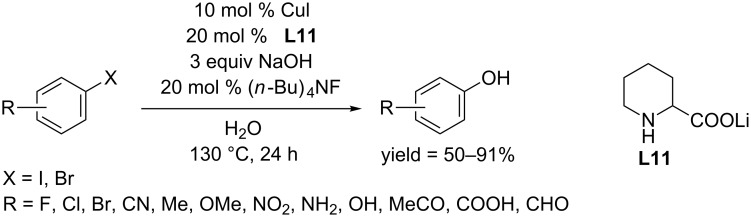
Hydroxylation of aryl halides using lithium pipecolinate as ligand.

Amino acids and its analogues are another type of N,O-bidentate ligand for copper catalyzed hydroxylation of aryl halides. In 2010, the Zhou group developed a CuI catalyzed protocol for hydroxylation of aryl iodides and bromides using lithium pipecolinate (**L11**) as ligand, yielding phenols in moderate to good yields (Scheme 20) [44]. The reaction proceeded in the presence of (*n*-Bu)_4_NF and NaOH. Notably, the reaction was carried out in water, avoiding the use of an organic solvent. In addition, a broad substrate scope was observed; some sensitive functional groups, such as carboxylic acid, aldehyde and cyano were well tolerated.

**Scheme 21:**
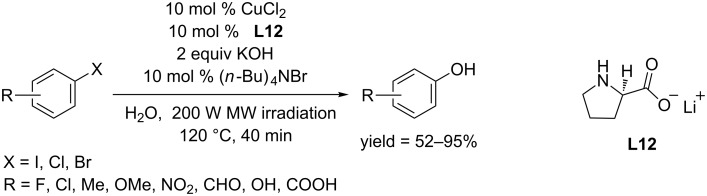
Hydroxylation of aryl halides using L-lithium prolinate.

In 2013, the Zhou group used lithium L-prolinate (**L12**) as ligand and developed a CuCl_2_ catalyzed protocol for converting aryl halides to phenols [45]. Aided by 200 W microwave irradiation, the conversion was accomplished within 40 minutes in the presence of (*n*-Bu)_4_NBr and KOH (Scheme 21). Using this reaction system, moderate yields of phenols could be obtained from both aryl iodides and aryl bromides. It is surprising that iodobenzenes bearing *para*-substitutes including methyl, methoxyl, cyano and bromo gave benzene as product rather than the corresponding phenols.

**Scheme 22:**
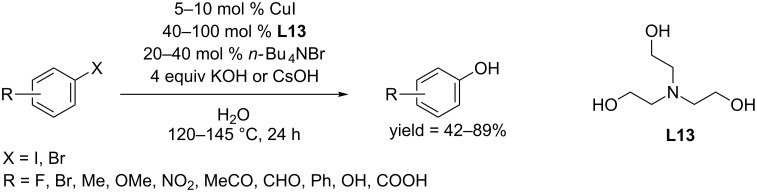
Hydroxylation of aryl halides using triethanolamine as ligand.

In 2014, the Wang group found triethanolamine (**L13**) could serve as ligand to synthesize phenols from aryl iodides and bromides in water using CuI as catalyst (Scheme 22) [46]. In the presence of KOH, aryl iodides containing both electron-donating and electron-withdrawing groups afforded the corresponding phenols in good to excellent yields. Aryl bromides were converted in good yields when treated with stronger base CsOH at 145 °C.

Although the ligand highly increased the catalytic activity of the copper catalyst, it was often used in large amount, making the process expensive and difficult in removing it. In this context, some ligand-free protocols have been developed by enhancing the reaction activity in some other ways.

**Scheme 23:**
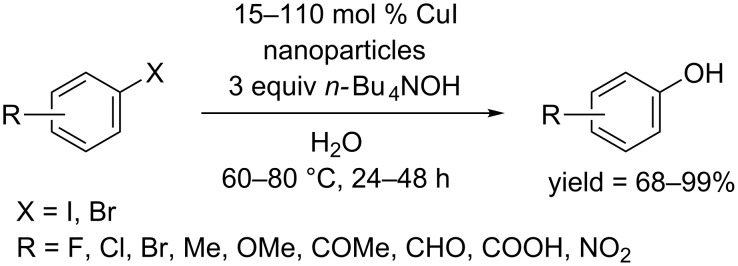
CuI-nanoparticle-catalyzed hydroxylation of aryl halides.

In 2011, the Xu and Feng group cooperatively reported the hydroxylation of aryl halides without ligand using CuI-nanoparticles as catalyst in the presence of (*n*-Bu)_4_NBr under very mild conditions [47]. CuI-nanoparticles could be reused with a slight loss of activity. The reaction was carried out in water at 60–80 °C, affording the phenols from aryl iodides and bromides in good to excellent yields (Scheme 23). In the case of aryl bromide, long reaction time (48 h) was required. 110 mol % of CuI-nanoparticles was needed for the complete conversion of aryl bromides bearing electron-donating groups. It's worth noting that the developed protocol could be easily applied to the synthesis of anilines and aryl thiols.

**Scheme 24:**
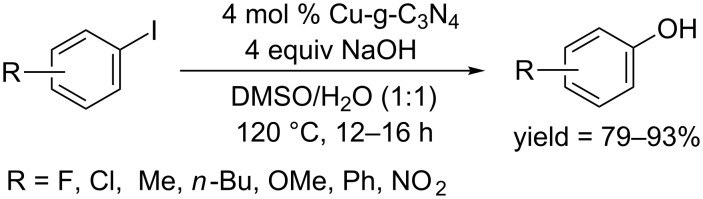
Cu-g-C_3_N_4_-catalyzed hydroxylation of aryl bromides.

In 2014, the Jiang and Han group used a copper-doped graphitic carbon nitride catalyst Cu-g-C_3_N_4_, which was prepared from urea and CuNO_3_, and developed a ligand free protocol for the synthesis of phenols from aryl iodides [48]. The conversion was achieved smoothly in the presence of NaOH in a mixed solvent of DMSO and H_2_O (Scheme 24).

As described above, both palladium and copper catalysts have shown promising effectivenesses in catalyzing the conversion of aryl halides to phenols. Generally, copper catalysts are more favorable for aryl bromides and aryl iodides as aryl chlorides require palladium catalysts. Except for special cases, a ligand is indispensable for the conversion. In the view of solvent, DMSO/H_2_O as mixed solvent or water as sole solvent are popular choices for the hydroxylation of aryl halides.

#### 1.2 Arenes as substrate

It has been a long time since C–H hydroxylation first appeared, however, in the beginning it often suffered from some drawbacks such as low selectivity and low yield. In 1991, the Fujiwara group showed the possibility of preparing phenol from benzene using Pd(OAc)_2_ as catalyst and molecular oxygen as oxidant, however, the reaction suffered from low yield (2.3%) [49–50]. In 1997, Seo and co-workers reported an iron-HPA (heteropoly acid)-complex-catalyzed protocol for oxidation of benzene to phenol [51]. In 2005, the Rybak-Akimova group reported that they used a stoichiometric amount of reactive iron complex [Fe(II)(BPMEN)(CH_3_CN)_2_](ClO_4_)_2_ to achieve *ortho*-hydroxylation of benzoic acid in the presence of H_2_O_2_, affording salicylic acid in low yields [52]. In the past decade, the selectivity and yield of C–H hydroxylation of arenes were highly improved by introduction of various directing groups.

**Scheme 25:**
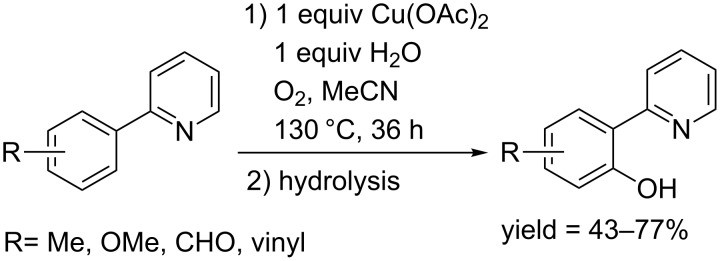
Cu(OAc)_2_-mediated hydroxylation of (2-pyridyl)arenes.

**1.2.1 Copper mediated C–H hydroxylation of arenes:** A breakthrough was made by Yu and co-workers in 2006. They reported a protocol for the hydroxylation of (2-pyridyl)arenes [53]. Under an atmosphere of oxygen, the reaction proceeded in the presence of a stoichiometric amount of Cu(OAc)_2_ and H_2_O in acetonitrile at 130 °C, and the resulting acetate gave phenols in moderate yields through a simple hydrolysis (Scheme 25). A mechanistic investigation revealed that the reaction proceeded via a radical-cation pathway. Notably, their protocol could be also applied in the chlorination and other C–H functionalization of (2-pyridyl)arenes. Together with this work, some other work on C–H acyloxylation provided an indirect pathway for the synthesis of phenols from arenes [54].

**Scheme 26:**
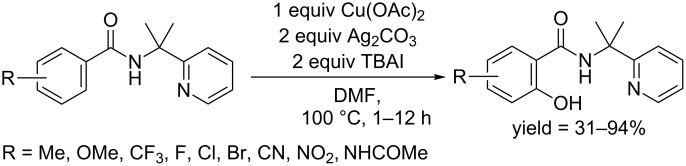
Removable pyridine moiety directed hydroxylation of arenes.

In 2014, Shi and co-workers designed a removable bidentate functional group, which could facilitate C–H hydroxylation of benzoic acids and heteroarenes [55]. An amidation reaction between benzoic acid and 2-(pyridine-2-yl)isopropylamine gave *N*-(2-(pyridine-2-yl)isopropyl)benzamides, which could be hydroxylated at the *ortho* position in moderate to excellent yields. The reaction was promoted by a stoichiometric amount of Cu(OAc)_2_ in the presence of Ag_2_CO_3_ and TBAI as additives in DMF at 100 °C (Scheme 26). Notably, their protocol was so efficient that the reactions could accomplish within 1 hour. The substrate scope showed benzamides bearing both electron-donating groups and electron-withdrawing groups were converted to the corresponding phenols. Moreover, heteroarenes, such as pyridine and thiophenes, could also give hydroxylated products under their conditions.

**Scheme 27:**
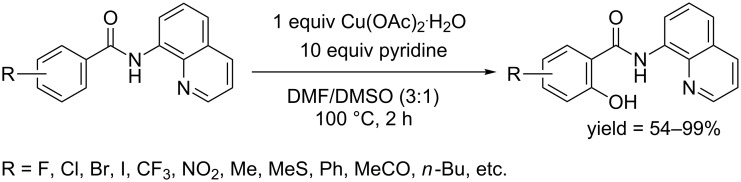
Removable quinoline moiety directed hydroxylation of arenes.

In 2016, Jana and co-workers used *N*-(8-quinolinyl)benzamides as starting materials, and *ortho*-hydroxylation occurred in the presence of a stoichiometric amount of Cu(OAc)_2_ and pyridine in DMSO/DMF (1:3) (Scheme 27) [56]. Hydroxylation of arenes could be accomplished within 2 hours. Both electron-donating groups and electron-withdrawing groups were well tolerated. The developed protocol could be also applied in the hydroxylation of aryl halides and aryl methyl ethers.

**Scheme 28:**
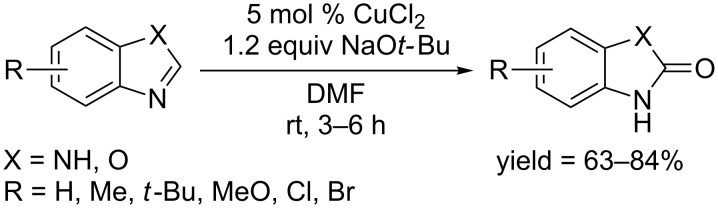
CuCl_2_ catalyzed hydroxylation of benzimidazoles and benzoxazoles.

Several examples using catalytic amounts of copper catalyst have also been reported. In 2012, the Lei group developed mild conditions using a catalytic amount of CuCl_2_ to convert heterocycles to the hydroxylated products in the presence of NaO*t-*Bu in the air at room temperature (Scheme 28) [57]. Muti-halogenated arenes also gave the corresponding phenols when treated under the above mentiond conditions. An mechanistic investigation showed that NaO*t-*Bu was a crucial reagent which played dual roles in affording the active CuO*t-*Bu from CuCl_2_, and deprotonation of heteroarenes before the formation of copper complex.

**Scheme 29:**
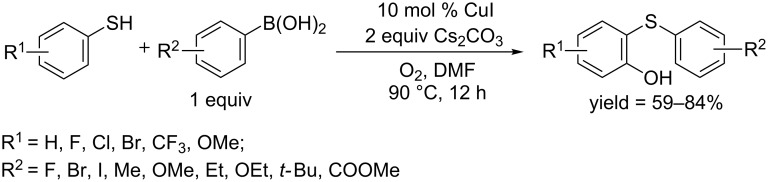
Disulfide-directed C–H hydroxylation.

In 2016, the Wang and Shi group reported a CuI-catalyzed C–H hydroxylation of thiophenols, in which disulfide directed the hydroxylation [58]. Using aryl thiol and arylboronic acid as starting materials, C–H hydroxylation and C–S coupling sequentially occurred in DMF in the presence of Cs_2_CO_3_ and molecular oxygen, affording 2-(phenylthio)phenols as final products (Scheme 29). A preliminary mechanistic study showed that molecular oxygen participated in the formation of the hydroxy group. This protocol was further applied to the synthesis of quinines.

**1.2.2 Palladium-catalyzed C–H hydroxylation of arenes:** Palladium catalysts were proved to be effective in catalyzing C–H hydroxylation of arenes and a variety of directing groups have been extensively studied and demonstrated to work as designed, thus providing plenty of strategies toward the preparation of phenols from arenes.

**Scheme 30:**
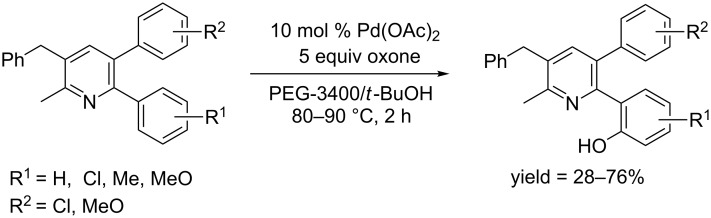
Pd(OAc)_2_-catalyzed hydroxylation of diarylpyridines.

**1.2.2.1 Pyridine and nitrogen containing functional groups as directing groups:** In 2008, Kim and co-workers synthesized a series of 2-arylpyridines, in which another aryl group was located at 3-position and a benzyl group at 5-position of the pyridine ring. Then they developed a Pd(OAc)_2_-catalyzed *ortho*-hydroxylation of the synthesized diarylpyridines [59]. Their protocol employed oxone as oxidant, allowing the conversion to complete within 2 hours in PEG-3400/*t*-BuOH at 80–90 °C (Scheme 30). By employing their protocol, *ortho*-hydroxyarenes were predominately formed. This method did not work with *ortho*-substituted arenes.

**Scheme 31:**
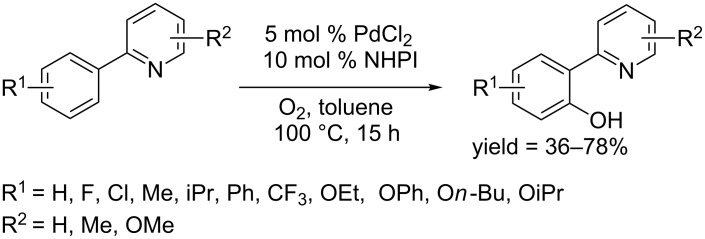
PdCl_2_-catalyzed hydroxylation of 2-arylpyridines.

In 2013, Jiao and co-workers reported a hydroxylation protocol for (2-pyridyl)arenes using PdCl_2_ and *N*-hydroxyphthalimide (NHPI) as catalyst and molecular oxygen as oxidant [60]. (2-Pyridyl)arenes were converted to the hydroxylated products in toluene at 100 °C (Scheme 31).

**Scheme 32:**
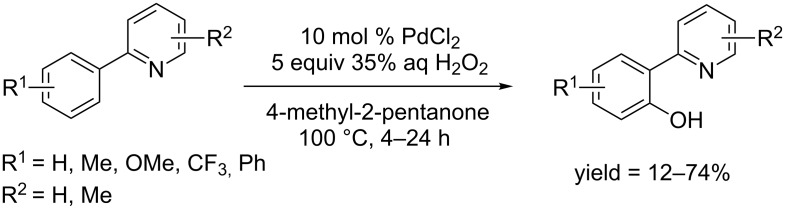
PdCl_2_-catalyzed hydroxylation of 2-arylpyridines.

In 2015, Itoh and co-workers employed PdCl_2_ as catalyst and the hydroxylation of arylpyridines was carried out in 4-methyl-2-pentanone at 100 °C in the presence of H_2_O_2_ (Scheme 32) [61]. In terms of reactivity, substituent at the *para*-position of the phenyl ring is more favored than at other positions.

**Scheme 33:**
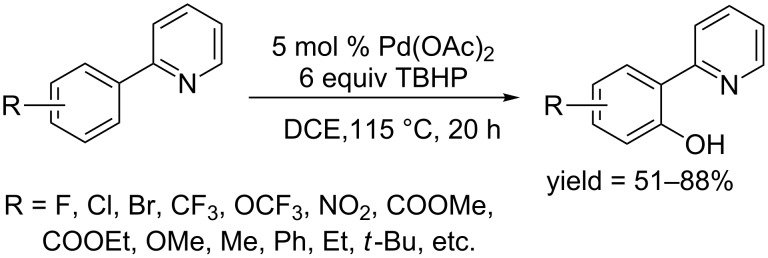
Pd(OAc)_2_-catalyzed hydroxylation of 2-arylpyridines.

In 2015, Sun and co-workers developed a Pd(OAc)_2_ catalyzed *ortho*-hydroxylation of 2-arylpyridines using *tert*-butyl hydroperoxide (TBHP) as oxidant [62]. The reaction was carried out at 115 °C in 1,2-dichloroethane (DCE), affording the corresponding phenols in moderate to good yields (Scheme 33). The reaction yield were lowered by adding a radical-trapping reagent, 2,2,6,6-tetramethyl-1-piperidinyloxy (TEMPO), indicating that radical HO·, which was generated from TBHP, may participate in the oxidation of the palladium complex from Pd(II) to Pd(IV).

**Scheme 34:**
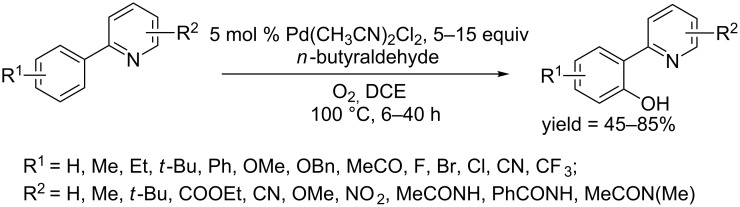
Pd(CH_3_CN)_2_Cl_2_-catalyzed hydroxylation of 2-arylpyridines.

In 2016, Guin and co-workers described a Pd(CH_3_CN)_2_Cl_2_ catalyzed C−H hydroxylation of 2-arylpyridines using molecular oxygen as oxidant [63]. The conversion was achieved in the presence of *n*-butyraldehyde in DCE at 100 °C, affording substituted 2-(pyridin-2-yl)phenols in good yields (Scheme 34). This protocol also involved radical species, such as an active acylperoxo-radical generated from oxygen and *n*-butyraldehyde. On both the phenyl ring or on the pyridine ring, electron-donating and electron-withdrawing groups were tolerated, respectively, although electron-deficient substrates required longer reaction time.

**Scheme 35:**
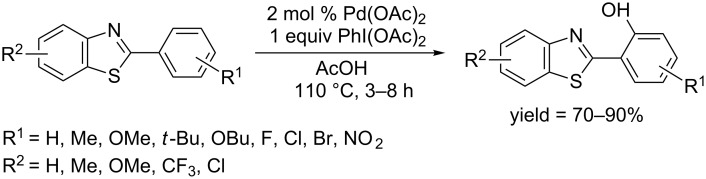
Pd(OAc)_2_-catalyzed hydroxylation of benzothiazolylarenes.

In 2013, Patel and co-workers succeeded in *ortho*-hydroxylation of 2-arylbenzothiazoles [64]. Directed by the benzothiazolyl group, Pd(OAc)_2_ catalyzed C–H hydroxylation occurred in acetic acid at 110 °C in the presence of diacetoxyiodobenzene (DIB) as oxidant (Scheme 35). Both electron-withdrawing groups and electron-donating groups are well tolerated and the protocol gave phenols in moderate to excellent yields. The analysis of the reaction performances indicated that the reaction proceeded through a Pd(II)/Pd(IV) catalytic cycle.

**Scheme 36:**
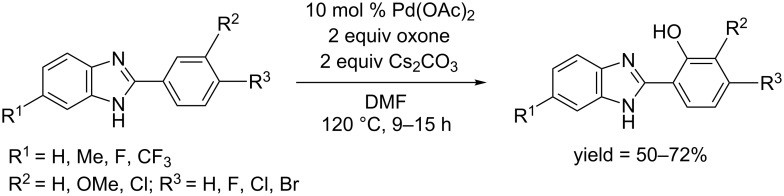
Pd(OAc)_2_ catalyzed hydroxylation of benzimidazolylarenes.

In 2014, a Pd(OAc)_2_ catalyzed protocol for the hydroxylation of 2-arylbenzimidazoles was developed by the Kamal and Nagesh group [65]. In their reaction system, oxone was used as oxidant, Cs_2_CO_3_ as base and DMF as solvent (Scheme 36). The reaction occurred at 120 °C and afforded the corresponding phenols in moderate yields. The catalytic system could also be used for alkoxylation of 2-arylbenzimidazoles when the solvent was replaced by aliphatic alcohols.

**Scheme 37:**
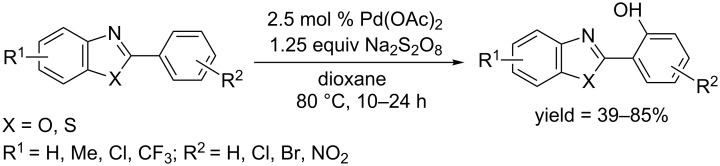
Dioxane mediated hydroxylation of 2-heteroarylarenes.

In 2015, Chakraborti and co-workers developed a Pd(OAc)_2_ catalyzed C–H hydroxylation of 2-benzoxazolyl- and 2-benzothiazolylarenes in the presence of Na_2_S_2_O_8_ as oxidant in 1,4-dioxane (Scheme 37) [66]. They also showed that other acyclic directing groups including azo, amide, anilide, carbamate and unsymmetrical urea, could also promote the *ortho*-hydroxylation of arenes. Remarkably, 1,4-dioxane not only served as the solvent, but also played an indispensable role in the oxidation of the palladium complex by generating hydroxyl radicals according to the proposed reaction mechanism.

**Scheme 38:**
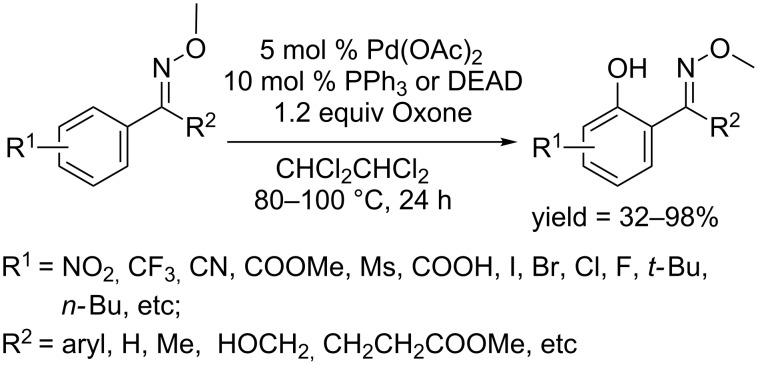
Hydroxylation of oxime methyl ester.

In 2015, Jiao and co-workers employed an oxime methyl ester as directing group and achieved the hydroxylation of arenes [67]. The reaction used Pd(OAc)_2_ as catalyst, PPh_3_ or DEAD as ligand and oxone as oxidant, affording the corresponding phenols in gratifying yields (Scheme 38). Exploration of the substrate scope showed that both electron-rich and electron-deficient substrates were tolerated, although the latter of which provided lower yields. The functional group R^2^ could vary from alkyl to aryl group, enabling the synthesis of diverse phenols. Moreover, the hydroxylated oxime could be readily converted to *o*-acylphenol or *o*-aminomethylphenol.

**Scheme 39:**
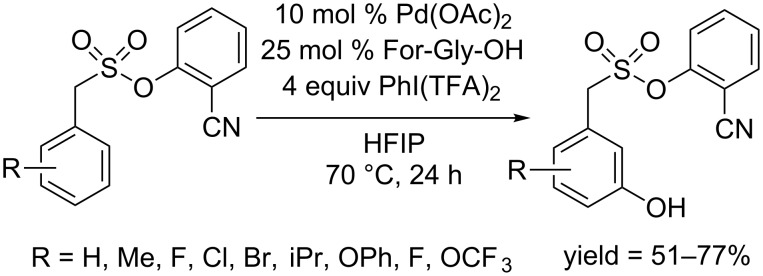
CN-directed *meta*-hydroxylation.

In 2016, Sunoj and co-workers disclosed the first *meta*-hydroxylation of arenes using a tethered -CN directing group [68]. The conversion proceeded at 70 °C in hexafluoro-2-propanol (HFIP) in the presence of Pd(OAc)_2_, PhI(TFA)_2_ and For-Gly-OH (Scheme 39). The substrate scope showed that both electron-donating and electron-withdrawing groups are tolerated, although electron-deficient arenes gave a bit lower yields. Notably, a replacement of PhI (TFA)_2_ with PhI(OAc)_2_ afforded acyloxylated products. A careful mechanism investigation revealed that HFIP participated in the catalytic cycle before the activation of C–H bond.

**Scheme 40:**
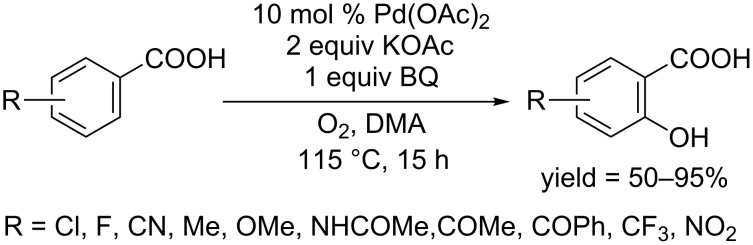
Pd(OAc)_2_-catalyzed hydroxylation of benzoic acids.

**1.2.2.2 Carboxylic acid, ketone and their derivatives as directing groups:** In 2009, the Yu group used Pd(OAc)_2_ and accomplished the direct *ortho*-hydroxylation of benzoic acid [69]. Their developed protocol used 10 mol % of Pd(OAc)_2_ as catalyst, and the reaction was carried out under the atmosphere of molecular oxygen in the presence of KOAc and 1,4-benzoquinone (BQ) as additives (Scheme 40). The mechanism investigation indicated that molecular oxygen was involved in the product forming step rather than reoxidation of Pd(0) as the reaction couldn't proceed with a stoichiometric amount of Pd(OAc)_2_ under argon.

**Scheme 41:**
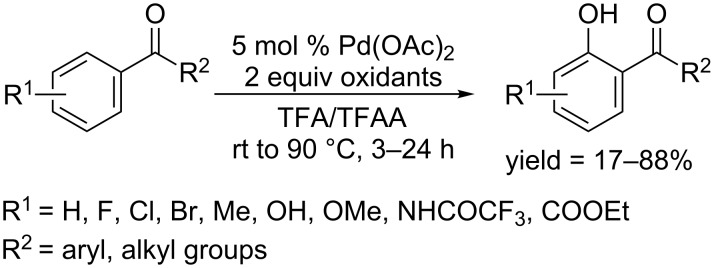
Pd(OAc)_2-_catalyzed hydroxylation of biaryl or aryl alkyl ketones.

In 2012, the Rao group and the Dong group simultaneously reported a palladium catalyzed *ortho*-hydroxylation of aryl ketones, and thus further broadened the directing groups of arene substrates [70–71]. Rao and co-workers found that both biaryl ketone and aryl alkyl ketones could be regioselectively hydroxylated in satisfying yields. The reaction proceeded in TFA/TFAA in the presence of Pd(OAc)_2_ as catalyst and several type of oxidants including selectfluor, PhI(OAc)_2_ and K_2_S_2_O_8_, respectively (Scheme 41). The regioselectivity was maintained in the presence of various functional groups. Further studies revealed that several functional groups such as esters, amides and sulfonamides could be added to the list of directing groups.

**Scheme 42:**
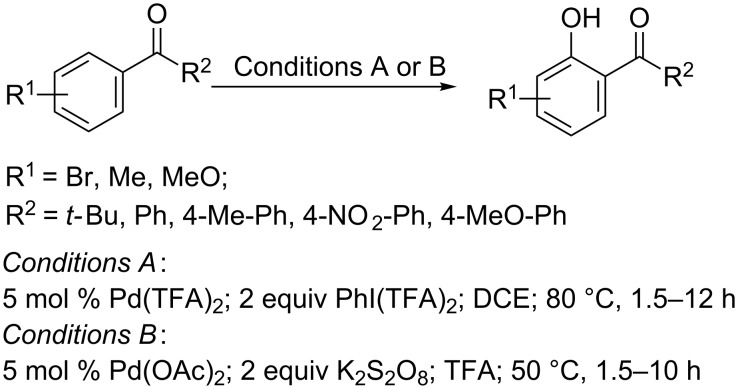
Pd(OAc)_2_ and Pd(TFA)_2_ catalyzed hydroxylation of aryl ketones.

The Dong group reported a similar work and developed two types of reaction conditions for the hydroxylation of aryl alkyl ketones. The conditions using PhI(TFA)_2_ as oxidant and DCE as the solvent were milder and more selective, while the conditions using K_2_S_2_O_8_ as oxidant and TFA as solvent were more reactive (Scheme 42). Interestingly, although the reaction gave the trifluoroacetates rather than phenols as products, phenols were readily obtained during the silica gel column chromatography.

**Scheme 43:**
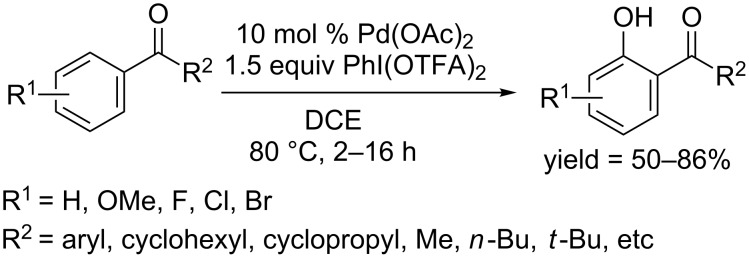
Pd(OAc)_2_ catalyzed hydroxylation of aryl ketones.

In 2013, an acid-free procedure for the regioselective hydroxylation of aryl ketones was reported by Kwong and co-workers [72]. They used Pd(OAc)_2_ as catalyst and PhI(OTFA)_2_ as oxidant, affording phenols in good yields in DCE at 80 °C (Scheme 43). The reaction system showed great efficiency as most reactions accomplished within 2 hours. Moreover, variable alkyl groups (R^2^) including cyclohexyl, cyclopropyl, butyl and *tert*-butyl, diversified the structure of the produced phenols.

**Scheme 44:**
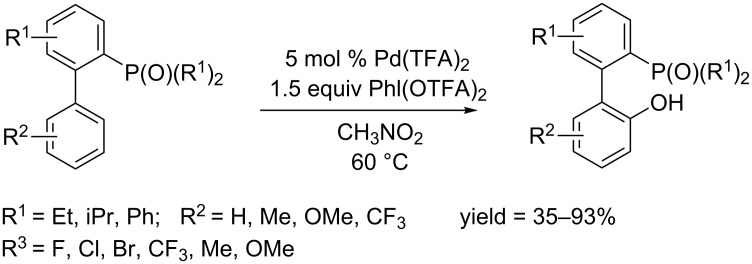
Pd(TFA)_2_-catalyzed hydroxylation of aryl phosphonates.

In 2013, Yang and co-workers reported the first phosphonate-directed hydroxylation of arenes for the synthesis of 2’-phosphorylbiphenyl-2-ol [73]. The reaction was catalyzed by Pd(TFA)_2_ in the presence of PhI(OAc)_2_ as oxidant (Scheme 44). Screening for directing groups found a series of dialkyl and diaryl phosphonates were compatible with the hydroxylation condtions, while monoalkyl phosphonate gave the phosphoryl lactone as product. Exploration of substrate scope showed that both electron-donating groups (such as Me, OMe) and electron-withdrawing groups (such as F, Cl, Br, CF_3_) are tolerable.

**Scheme 45:**
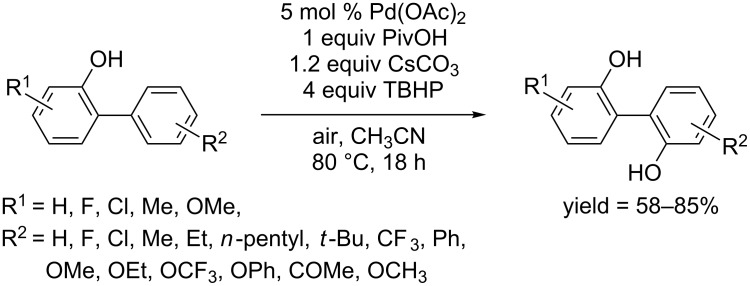
Hydroxy group directed hydroxylation.

**1.2.2.3 Phenol as directing groups:** In 2016, the Zhang and Fan group described the first phenolic moiety directed hydroxylation. Pd(OAc)_2_ catalyzed hydroxylation of [1,1’-biphenyl]-2-ols using TBHP as oxidant in acetonitrile (Scheme 45) [74]. The reaction predominantly afforded biphenols as product, rather than dibenzofurans through an intramolecular transformation. Similar with Sun's work in 2015 [62], this conversion was also considered to proceed through an oxidation of Pd(II) to Pd(IV) by radical HO·. Under the optimum conditions, a broad scope of functional groups was well tolerated.

**Scheme 46:**
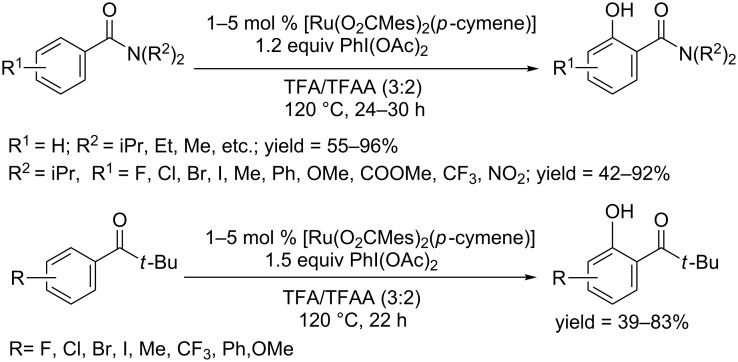
[Ru(O_2_CMes)_2_(*p*-cymene)] catalyzed hydroxylation of benzamides and aryl ketones.

**1.2.3 Ruthenium catalyzed C–H hydroxylation of arenes:** In 2012, Ackermann and co-workers used [Ru(O_2_CMes)_2_(*p*-cymene)] as catalyst to achieve the C–H bond oxygenation of *N*-substituted benzamides in TFA/TFAA. The reaction proceeded in the presence of PhI(OAc)_2_ as oxidant at 120 °C (Scheme 46) [75]. A broad scope of functional groups could be tolerated, giving a variety of phenols. Further studies found that this protocol could also be readily applied in the hydoxylation of aryl ketones [76]. Competition experiments showed that an electron-donating group was more feasible for this conversion than an electron-withdrawing group.

**Scheme 47:**
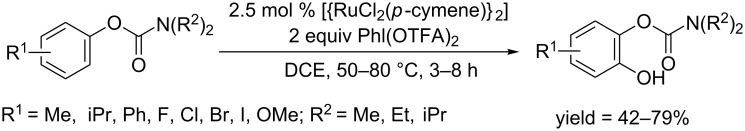
[RuCl_2_(*p*-cymene)]_2_-catalyzed hydroxylation of benzamides and carbamates.

In 2013, the Ackermann group used another ruthenium catalyst, [RuCl_2_(*p*-cymene)]_2_ and developed a hydroxylation protocol for carbamates [77]. The hydroxylation occurred in DCE in the presence of PhI(OTFA)_2_ (Scheme 47). In intermolecular competition experiments, among amide, carbamate and ester, amide showed the highest efficiency while ester showed the lowest efficiency in directing the C–H hydroxylation. Moreover, this protocol could be easily applied in the hydroxylation of anisoles.

**Scheme 48:**
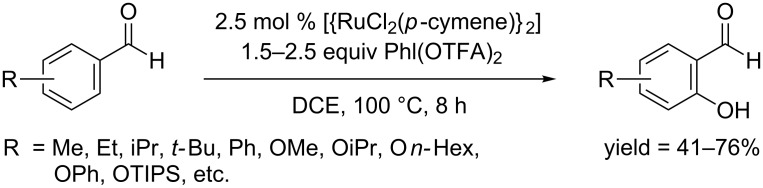
[RuCl_2_(*p*-cymene)]_2_ catalyzed hydroxylation of benzaldehydes.

They further successfully applied this catalytic system to the hydroxylation of benzaldehydes, affording 2-hydroxylbenzaldehydes in moderate yields (Scheme 48) [78]. In terms of substrate scope, both electron-rich and electron-deficient benzaldehydes provided comparable yields. Intermolecular competition experiments showed that weakly coordinating aldehydes gave lower yields under the same conditions when compared with amides and ketones.

**Scheme 49:**
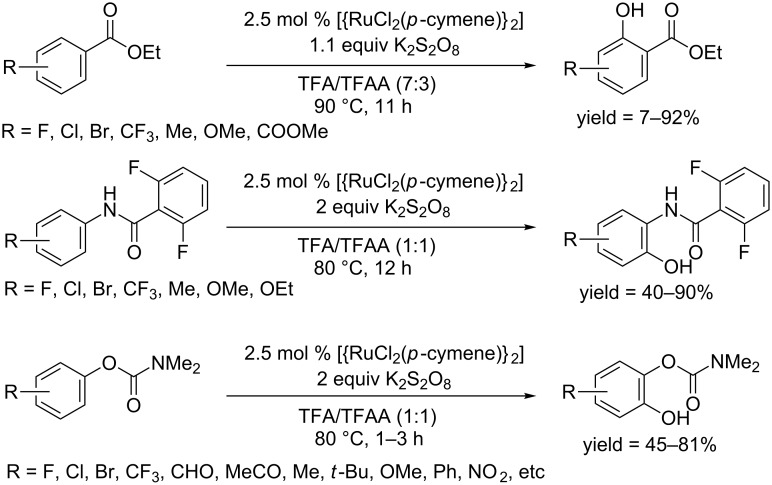
[RuCl_2_(*p*-cymene)]_2_ catalyzed hydroxylation of ethyl benzoates, benzamides and carbamates.

The Rao group developed a catalytic system of [RuCl_2_(*p*-cymene)]_2_ and K_2_S_2_O_8_ in TFA/TFAA, which promoted the hydroxylation of ethyl benzoates, benzamides and carbamates (Scheme 49). They reported the first *ortho*-hydroxylation of ethyl benzoates in 2012 [79]. Both electron-rich and electron-deficient ethyl benzoates were readily converted to the corresponding hydroxylated products. In 2013, they further applied this protocol to the hydroxylation of *N*-aryl-2,6-difluorobenzamides [80]. The *ortho*-, *meta*-, and *para*-substituent groups, as well as the electron-withdrawing groups (such as halides, CF_3_, ester, etc.) and electron-donating functional groups (such as methyl, methoxy, etc) were well tolerated. In 2014, the tolerance of this protocol for carbamates and esters was studied [81]. Carbamates were compatible with this protocol, however, esters gave lower yields. A replacement of [RuCl_2_(*p*-cymene)]_2_ with Pd(OAc)_2_ gave the hydroxylated carbamates at room temperature although yields were lowered.

**Scheme 50:**
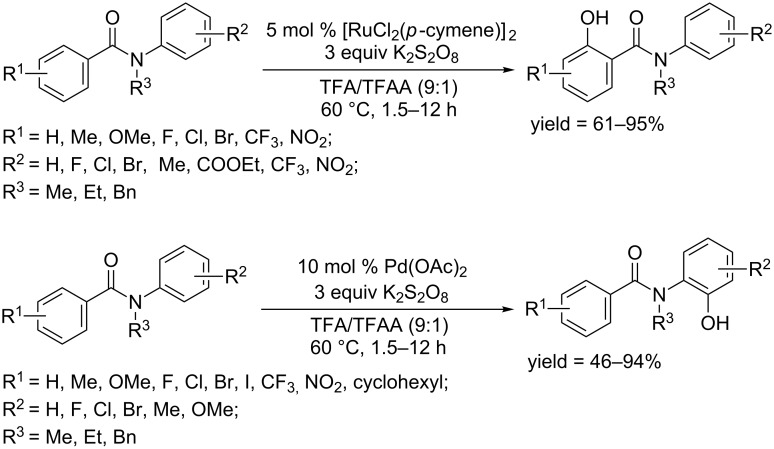
Different regioselective *ortho*-hydroxylation.

In 2016, the Rao group described a regioselective hydroxylation protocol for benzanilides [82]. Two phenyl rings of *N*-alkylbenzanilides could be differentiated in the *ortho-*hydroxylation by choosing different catalysts (Scheme 50). In the catalytic system consisting of [RuCl_2_(*p*-cymene)]_2_ and K_2_S_2_O_8_, the C–H hydroxylation occurred at the position *ortho* to the carbonyl group. Different regioselective hydroxylation was observed when [RuCl_2_(*p*-cymene)]_2_ was replaced with Pd(OAc)_2_, by which C–H hydroxylation predominantly occurred at the position *ortho* to the aniline group. A mechanism investigation showed that the different regioselectivity was controlled by steric and electronic effects.

**Scheme 51:**
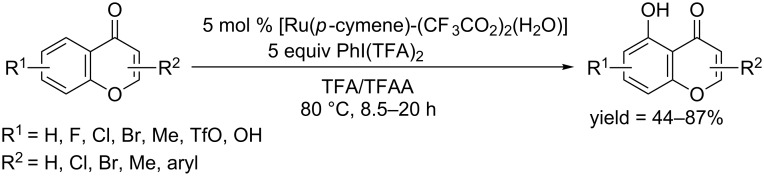
Ruthenium-complex-catalyzed hydroxylation of flavones.

In 2015, Hong and co-workers developed a C–H hydroxylation protocol for flavones; their work is of importance because the hydroxy group has a great impact on the biological activities of 5-hydroxyflavonids [83]. Starting from [RuCl_2_(*p*-cymene)]_2,_ Ag_2_CO_3_ and CF_3_COOH, they prepared a ruthenium complex, which could achieve the regioselective hydroxylation of flavones at 80 °C in TFA/TFAA in the presence of PhI(TFA)_2_ as oxidant (Scheme 51).

**Scheme 52:**
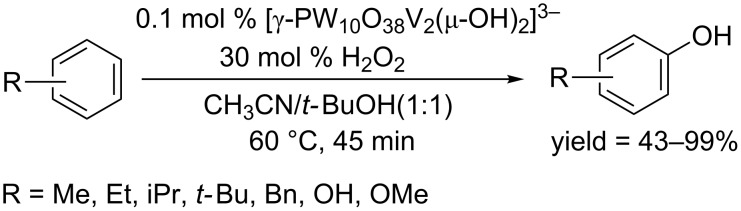
Vanadium-catalyzed hydroxylation of arenes.

**1.2.4 Vanadium mediated C–H hydroxylation of arenes:** In 2012, Mizuno employed divanadium-substituted phosphotungstate, and accomplished the direct hydroxylation of structurally simple arenes to phenols in the presence of H_2_O_2_ [84]. The reaction occurred at 60 °C in CH_3_CN/*t-*BuOH (1:1) and afforded phenols in good to excellent yields (Scheme 52). In most cases, *para*-hydroxylation predominantly occurred, showing good regioselectivity. This protocol is suitable for hydroxylation of simple arenes as no particular directing group was required.

**Scheme 53:**
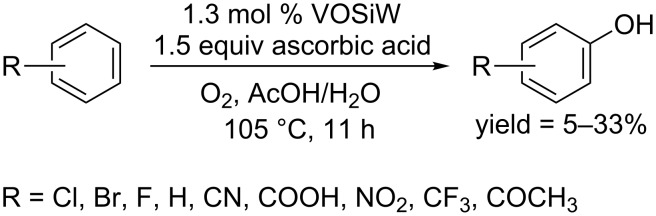
VOSiW-catalyzed hydroxylation of arenes.

In 2015, the Huang group prepared a type of vanadium catalysts supported on N-doped carbon materials (VOSiW), which showed catalytic activity to convert electron-deficient arenes to phenols, however, the yields and selectivity were not very satisfying (Scheme 53) [85].

### 2 Transition-metal-catalyzed synthesis of aryl thiols

Traditional methods for the synthesis of aryl thiols include the Leukart thiophenol synthesis [86], the Newman–Kart reaction [87–88] and the Schonberg reaction [89]. These methods often require multiple steps and/or harsh conditions. The reduction of phenylsulfonic chloride and disulfide can also afford aryl thiols, but the preparation of these specific precursors often needs laborious work.

The direct nucleophilic substitution of aryl halides with sodium alkyl sulfate was considered a simple strategy for the synthesis of aryl thiols [90–92]. However, these non-catalyzed conversions require excess amounts of sodium alkyl sulfate, high temperatures and toxic organic solvents, and thus limited the substrate scope and wide application.

As mentioned above, the development of transition-metal-catalyzed C–O coupling reaction stimulated the emergence of new protocols for phenol synthesis. However, a rapid development of new methods for aryl thiol preparation was not observed. There may be two reasons which block the development of synthetic methods for aryl thiols: (1) Traditionally, sulfur was considered poisonous to transition metal catalysts [93–94]; (2) aryl thiols are very reactive forming intermolecular or intramolecular sulfide and disulfide compounds, so that it is difficult to isolate aryl thiols as final product. For example, many reactions use aryl thiols as active intermediates to form benzothiazoles from aryl halides [95–96].

The first report of a transition-metal-catalyzed synthesis of aryl thiol appeared in 1985 [97], but there was no report until this century. Most of the known methods involve a two-step strategy. More recently, a few examples of single-step syntheses of aryl thiols have been reported.

#### 2.1 Two step strategy for the mercaptolization of aryl halides

**Scheme 54:**
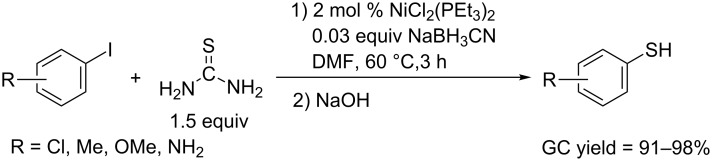
Synthesis of aryl thiols using thiourea as thiol source.

In 1985, Tiecco and co-workers succeeded in a Nickel catalyzed thiolation of aryl iodides with thiourea in DMF. Aryl iodides firstly reacted with thiourea in the presence of bis(triethylphosphine)nickel(II) chloride and sodium cyanoborohydride as catalyst precusor, and afforded aryl isothiuronium iodide, which could be further converted to aryl thiol through an alkali hydrolysis (Scheme 54). Compared with non-catalyzed methods, this protocol has several advantages, such as low reaction temperature (60 °C), broader substrate scope including OMe and NH_2_-substituted iodobenzenes, and higher yields up to 98% (GC determined). In 2010, the Qi group reported a similar protocol to achieve the coupling of aryl iodides and thiourea by CuI/L-proline-catalyzed reaction in the presence of Cs_2_CO_3_ as base in DMSO [98].

**Scheme 55:**
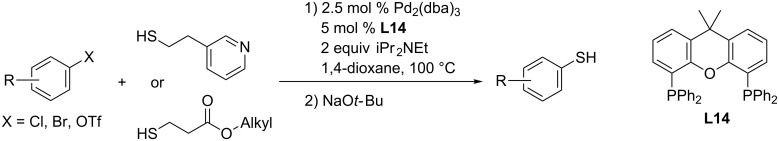
Synthesis of aryl thiols using alkyl thiol as thiol source.

In 2004, the Itoh group demonstrated that Pd_2_(dba)_3_/Xantphos (**L14**) could catalyze the coupling of aryl halides and aryl or alkyl thiols in the presence of iPr_2_Net (Scheme 55) [99]. They showed that the coupled products, aryl pyridinethyl thioether and aryl alkyloxycarbonylethyl thioether, could be converted to aryl thiols under specified conditions, respectively.

**Scheme 56:**
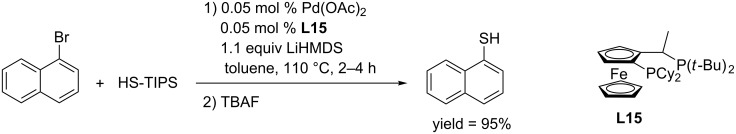
Synthesis of 1-thionaphthol using HS-TIPS as thiol source.

In 2010, the Hartwig group developed a Pd(OAc)_2_/CyPF-*t*-Bu (**L15**) catalyzed coupling of aryl bromides and TIPS-SH, where the reaction was carried out in the presence of lithium bis(trimethylsilyl)amide (LiHMDS) in toluene at 110 °C (Scheme 56) [100]. The coupled product of 1-bromonaphthalene and TIPS-SH could be readily converted to 1-thionaphthol when treated with TBAF.

**Scheme 57:**
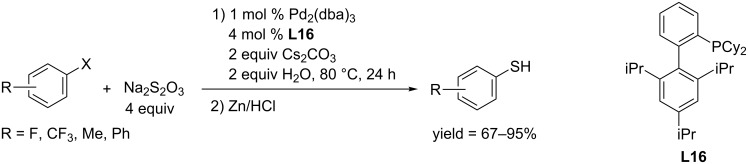
Synthesis of aryl thiols using sodium thiosulfate as thiol source.

In 2011, the Fu and Guo group reported that Pd(OAc)_2_/X-Phos (**L16**) and Pd_2_(dba)_3_/X-Phos-catalyzed thiolation of aryl bromide and chloride using sodium thiosulfate as thiol source [101]. The coupling reaction proceeded in water in the presence of Cs_2_CO_3_, and first gave aryl thiosulfate, which was further reduced by Zn/HCl in the following step to provide aryl thiols (Scheme 57). Their developed protocol could convert aryl bromide, aryl chloride and aryl trifluoromethanesulfonate to the corresponding aryl thiols in moderate to excellent yields.

**Scheme 58:**
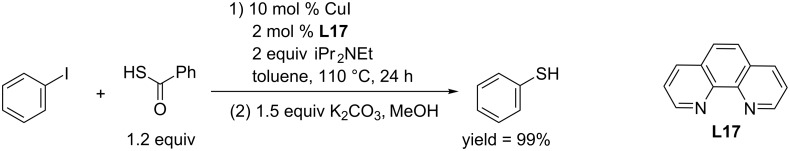
Synthesis of thiophenol using thiobenzoic acid as thiol source.

In comparison to palladium catalysts, copper catalyzed thiolation of aryl halides have been extensively studied. In 2006, the Sawada group reported that aryl iodides could couple with thiobenzoic acid in the presence of a copper catalyst and 1,10-phenanthroline (**L17**), affording *S*-aryl thiocarboxylates in excellent yields [102]. The coupled product was converted to aryl thiols in quantitative yield when treated with K_2_CO_3_ (Scheme 58).

**Scheme 59:**
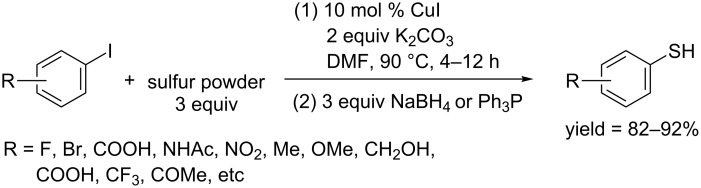
Synthesis of aryl thiols using sulfur powder as thiol source.

In 2009, Ma and co-workers unprecedentedly used simple and economical sulfur powder as thiol source and developed an effective method for the synthesis of aryl thiols. They demonstrated that simple sulfur powder could couple with aryl iodides at 90 °C in the presence of CuI as catalyst to afford biaryl disulfides and polysulfides, which could be further converted to aryl thiols through a followed reduction using NaBH_4_ or PPh_3_ (Scheme 59) [103]. A wide range of functional groups including methoxy, hydroxy, acyl, carboxy, amide, bromo and trifluoromethyl were tolerated in this process. They also showed the application of the developed protocol in the synthesis of aryl alkyl sulfides via a “one-pot reaction”.

**Scheme 60:**
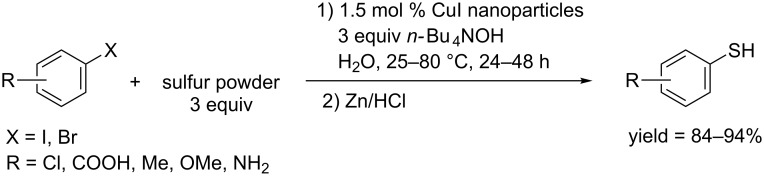
CuI-nanoparticles catalyzed synthesis of aryl thiols.

In 2011, the Xu and Feng group applied CuI-nanoparticles to synthesize aryl thiols through a coupling reaction of aryl halides and sulfur powder followed by a reduction using Zn/HCl (Scheme 60) [47]. The reaction proceeded in water in the presence of *n*-Bu_4_NOH, making the reaction green. Both aryl iodides and activated aryl bromides could give moderate to excellent yields.

#### 2.2 Single step strategy for the mercaptolization of aryl halides

Transition-metal-catalyzed methods provides various thiolation protocols, which allow an access to aryl thiols. However, all these methods described above require two steps, causing more labor both in the laboratory and industry. In this context, one step conversions were desirable.

**Scheme 61:**
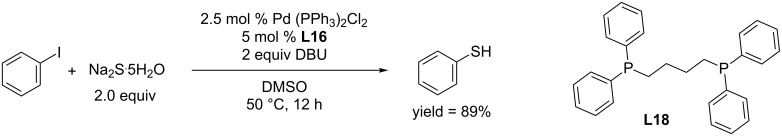
Synthesis of aryl thiols using Na_2_S·5H_2_O as thiol source.

In 2013, in the study of the synthesis of (*Z*)-3-arylthioacrylic acids and thiochromenones, Lee and co-workers found that 89% yield of thiophenol could be directly obtained by Pd (PPh_3_)_2_Cl_2_/dppb (**L18**) catalyzed coupling reaction of iodobenzene and Na_2_S·5H_2_O in the presence of DBU (Scheme 61) [104].

**Scheme 62:**
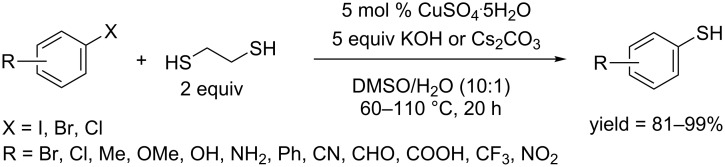
Synthesis of aryl thiols using 1,2-ethanedithiol as thiol source.

In 2015, on the basis of a successful application of ethylene glycol in a phenol synthesis [39], the Chae group developed a single-step protocol for the direct synthesis of aryl thiols. The protocol employed CuSO_4_·5H_2_O as the catalyst and KOH or Cs_2_CO_3_ as bases, and could convert aryl iodides, and aryl bromides bearing electron-withdrawing groups to the corresponding aryl thiols in good to excellent yields (Scheme 62) [105]. During the reaction, neither disulfide nor sulfide was formed. A simple investigation showed that aryl halides may first couple with 1,2-ethanedithiol and the coupled product was converted in situ to aryl thiols through C–S bond cleavage by an intramolecular nucleophilic substitution. The protocol tolerated a broad range of functional groups such as amino, hydroxy, trifluoromethyl, ester, carboxy and formyl groups.

As described above, although it seems more difficult to develop an effective protocol for the synthesis of aryl thiols than for phenols, some pioneering work showed great improvements in this field. On the other hand, to the best of our knowledge, there is no report on the synthesis of aryl thiols through C–H activation.

## Conclusion

In conclusion, tremendous progress has been made towards the synthesis of phenols and aryl thiols through transition-metal-catalyzed coupling reactions. However, there is still much space for improvement, and therefore the research in this field will be continued. In terms of the synthesis of phenols, since transition-metal-catalyzed hydroxylations of aryl halides have been well established, further efforts should be made in the C–H hydroxylation of arenes, allowing more types of arenes to be directly hydroxylated affording phenols. On the other hand, since *ortho*-hydroxylation has been extensively studied, remote direction of C–H hydroxylation can be another orientation for further developments. In terms of the synthesis of aryl thiols, the direct synthesis of aryl thiols using simple thiol sources and C–H mercaptolization of arenes would be desired as future direction.
